# Reversal of evoked gamma oscillation deficits is predictive of antipsychotic activity with a unique profile for clozapine

**DOI:** 10.1038/tp.2016.51

**Published:** 2016-04-19

**Authors:** M R Hudson, G Rind, T J O'Brien, N C Jones

**Affiliations:** 1 Department of Medicine, Royal Melbourne Hospital, Melbourne Brain Centre, University of Melbourne, Parkville, VIC, Australia

## Abstract

Recent heuristic models of schizophrenia propose that abnormalities in the gamma frequency cerebral oscillations may be closely tied to the pathophysiology of the disorder, with hypofunction of *N*-methyl-d-aspartate receptors (NMDAr) implicated as having a crucial role. Prepulse inhibition (PPI) is a behavioural measure of sensorimotor gating that is disrupted in schizophrenia. We tested the ability for antipsychotic drugs with diverse pharmacological actions to (1) ameliorate NMDAr antagonist-induced disruptions to gamma oscillations and (2) attenuate NMDAr antagonist-induced disruptions to PPI. We hypothesized that antipsychotic-mediated improvement of PPI deficits would be accompanied by a normalization of gamma oscillatory activity. Wistar rats were implanted with extradural electrodes to facilitate recording of electroencephalogram during PPI behavioural testing. In each session, the rats were administered haloperidol (0.25 mg kg^−1^), clozapine (5 mg kg^−1^), olanzapine (5 mg kg^−1^), LY379268 (3 mg kg^−1^), NFPS (sarcosine, 1 mg kg^−1^), d-serine (1800 mg kg^−1^) or vehicle, followed by the NMDAr antagonists MK-801(0.16 mg kg^−1^), ketamine (5 mg kg^−1^) or vehicle. Outcome measures were auditory-evoked, as well as ongoing, gamma oscillations and PPI. Although treatment with all the clinically validated antipsychotic drugs reduced ongoing gamma oscillations, clozapine was the only compound that prevented the sensory-evoked gamma deficit produced by ketamine and MK-801. In addition, clozapine was also the only antipsychotic that attenuated the disruption to PPI produced by the NMDAr antagonists. We conclude that disruptions to evoked, but not ongoing, gamma oscillations caused by NMDAr antagonists are functionally relevant, and suggest that compounds, which restore sensory-evoked gamma oscillations may improve sensory processing in patients with schizophrenia.

## Introduction

Schizophrenia is a complex neuropsychiatric disorder characterized by the triumvirate of positive, negative and cognitive symptoms. The primary medications used to treat this disorder, termed antipsychotics, are effective at addressing the positive symptoms including hallucinations and delusions, but generally lack efficacy against the emotional and cognitive disturbances.^1^ The atypical antipsychotic clozapine appears to have a unique spectrum of activity, providing therapeutic benefits in cases of treatment-resistant schizophrenia and also addressing cognitive complaints,^2, 3^ although there remains a speculation about this. Overall, there is a pressing need to develop new therapies that target cognitive symptoms in schizophrenia, which are now recognized as core features of the disease and the best predictor of long-term disability and functional outcome.^4^

Although the underlying pathophysiology of the disorder is incompletely understood, deficient signalling through *N*-methyl-d-aspartate receptors (NMDAr) is a prominent candidate mechanism.^5^ A primary observation driving this theory is that NMDAr antagonists such as ketamine and phencyclidine reproduce many of the clinical features of schizophrenia in healthy individuals and exacerbate pre-existing symptoms in schizophrenia patients.^6, 7^ This evidence is supported with a range of molecular and genetic observations linking NMDAr disruption to schizophrenia, and has subsequently led to the targeted testing of molecules which enhance the function of the NMDA receptor, some of which have shown promise in early trials.^8^ There is also a large body of experimental work using acute pharmacological NMDAr antagonism as an animal model of schizophrenia,^9^ and these drugs produce a range of behavioural effects, including cognitive dysfunction and sensory deficits, which are theoretically relevant to the perturbations of a brain of someone with schizophrenia.

Cortical gamma frequency oscillations (30–80 Hz) are intimately linked with a variety of cognitive processes, including sensory gating, perception, working memory and attention^10, 11^ and these brain rhythms have received much attention in schizophrenia research,^12^ as the same cognitive processes driven by gamma oscillations are known to be disrupted in the disorder. For example, ongoing or spontaneous gamma oscillations have been shown to be elevated in schizophrenia patients,^13, 14, 15^ whereas in apparent contradiction, other studies demonstrate reductions in stimulus-evoked gamma responses.^11, 16, 17^ Considering the relevance of gamma oscillations to sensory and cognitive processing, and the observations of disturbed gamma oscillations in patients, it is feasible that these electrophysiological abnormalities directly cause certain aspects of schizophrenia symptomatology. If this is the case, then identification of gamma deficits may be useful for diagnostic purposes and disease biomarkers, as well as for screening novel antipsychotic compounds that address cognitive symptoms of schizophrenia.

In addition to producing behavioural abnormalities, NMDAr antagonists disrupt cortical gamma frequency oscillations (30–80 Hz) in rodents and humans in a manner similar to that observed in patients with schizophrenia. They dose-dependently increase the power of ongoing gamma oscillations,^18, 19, 20^ and reduce evoked gamma oscillations elicited by sensory stimuli.^21, 22, 23, 24^ The acute NMDAr antagonist model therefore provides an opportunity to investigate the relationships between different neuronal oscillatory consequences of NMDAr hypofunction and the behavioural states which are induced by NMDAr antagonists. We previously tied NMDAr-mediated gamma abnormalities to deficits in sensorimotor gating using a combined electroencephalogram (EEG)-behaviour study assessing prepulse inhibition (PPI), a robust behavioural measure recognized to be abnormal in patients with schizophrenia.^25^ We found that gamma oscillations evoked by the behavioural task were significantly reduced following the treatment with NMDAr antagonists concurrently with impaired behavioural performance.^23^ Here, we sought to establish the predictive validity of this model, hypothesizing that the ability of drugs to attenuate NMDAr antagonist-induced PPI deficits would be related to their ability to attenuate NMDAr antagonist-induced disruptions to gamma oscillations. To test this hypothesis, we concurrently measured behaviour and electrophysiology (both ongoing activity and sensory-evoked oscillations), and tested the ability of a range of clinical and preclinical antipsychotic drugs to mitigate deficits induced by NMDAr antagonists. The test drugs included the typical antipsychotic, haloperidol; atypical antipsychotics, clozapine and olanzapine; LY379268, a selective agonist at mGluR_2/3_ (ref. 26), which can oppose some relevant actions of acute NMDAr antagonism, such as the enhanced locomotor activity^27^ and excessive cortical glutamate release^28^ observed following administration of NMDAr antagonists; NFPS (sarcosine), a selective inhibitor of GLYT1, which acts to prolong the synaptic activity of the NMDAr co-agonist glycine;^29^ and d-serine, an agonist at the glycine modulatory site.

## Materials and methods

### Animals

Adult (aged 12–16 weeks) male Wistar rats (250–350 g) were purchased from ARC (Perth, WA, Australia) and individually housed at the Melbourne Brain Centre, University of Melbourne. The facility was maintained on a 12 h light/dark cycle (0730–1930 h) with food (standard rat chow) and water available *ad libitum*. All the experimental procedures were approved by the University of Melbourne Animal Ethics Committee (#1011868).

### Electrode implantation surgery

Each animal was surgically implanted with brass extradural recording electrodes (PlasticsOne, BioScientific, NSW, Australia), as previously described.^18^ Briefly, the animals were anaesthetized with isoflurane and placed in a stereotaxic frame. A single midline incision was made over the scalp and six small holes drilled through the skull at 2 mm anterior and 2 mm lateral to bregma bilaterally (active electrodes), 2 mm posterior and 2 mm lateral to bregma bilaterally (ground electrodes), and 2 mm anterior and 2 mm lateral to lambda bilaterally (reference electrodes).^30^ We focussed on recording EEG at the somatosensory cortex to keep consistency with our previous studies.^23^ The electrodes were gently screwed into the holes, and the free ends inserted into a plastic multi-channel electrode pedestal (PlasticsOne, Bioscientific), which was secured to the skull using dental cement.

### Experimental protocol

**Figure 1:**
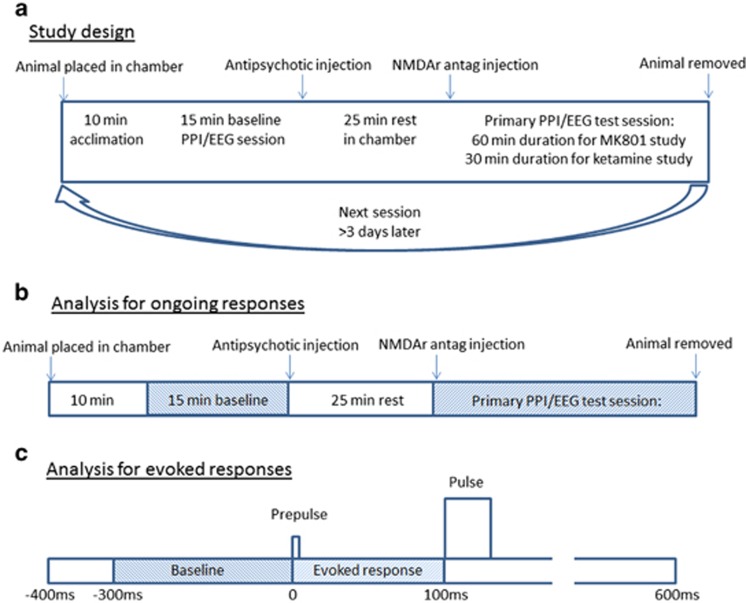
Study design and electrophysiological analyses. (**a**) Detailed study design. The animals are placed into the PPI chamber for 10 min acclimation, followed by 15 min baseline acquisition of PPI and EEG. They are then injected with antipsychotic, placed back in the chamber for 25 min, at which time they are injected with NMDAr antagonist. The primary PPI/EEG data acquisition phase then begins, continuing for 60 min in MK801-treated rats or 30 min for ketamine-treated rats. The process is repeated at least 3 days later, with injection of a different antipsychotic. (**b**) Schematic illustration of the ongoing gamma power analysis. The EEG data are split into 2-s epochs, which each undergo Fast Fourier Transformation. The power in 30–80 Hz (gamma) frequency range is averaged each 60 s (30 epochs). The data generated from the primary PPI/EEG period are then normalized to the average power in the baseline period (=100%) for each trial. (**c**) Schematic illustration of the evoked gamma power analysis. The epochs are extracted for all prepulse+pulse trials. These epochs incorporate the period −400 ms to +600 ms relative to the onset of the prepulse occurring in the primary period. The extracted epochs are subjected to Morlet wavelet decomposition. All the values between 30 and 80 Hz occurring between 0 and 100 ms relative to the onset of the prepulse are averaged and then normalized (baseline-corrected) to values between 30 and 80 Hz occurring between −300 and 0 ms relative to the prepulse. EEG, electroencephalogram; NMDAr, *N*-methyl-d-aspartate receptor; PPI, prepulse inhibition.

Each experiment consisted of simultaneous recording of EEG and measurement of PPI. At the start of each session, each rat was placed inside the PPI chamber (San Diego Instruments, San Diego, CA, USA) and the electrode headpiece attached to a six-channel cable. The PPI sessions began with a 10-min acclimatization period followed by a 15-min baseline PPI session. The animals were then injected subcutaneously with antipsychotic and immediately returned to the PPI chamber for 25 min, at which time they were again removed and injected subcutaneously with NMDAr antagonist. They were then placed back in the chamber and exposed to another period of continuous PPI testing (of variable duration—see below). This period constituted our primary data acquisition. We used two cohorts of different animals: the first cohort (*n*=6) received haloperidol (0.25 mg kg^−1^), clozapine (5 mg kg^−1^), LY379268 (3 mg kg^−1^), NFPS (1 mg kg^−1^), d-serine (1800 mg kg^−1^) or 0.9% saline followed by MK-801 (0.16 mg kg^−1^); the second (*n*=6) received haloperidol (0.25 mg kg^−1^), clozapine (5 mg kg^−1^), olanzapine (5 mg kg^−1^) or 0.9% saline followed by ketamine (5 mg kg^−1^). For the MK801 study, the primary period of PPI testing was 60 min, whereas due to the differing pharmacokinetics of the NMDAr antagonists, the primary period of testing for the ketamine study was 30 min. The treatments were given to each animal in a random order and at least 3 days passed between subsequent sessions. All the animals in a given cohort received all the drug combinations, allowing for our repeated-measures analysis—if an animal did not complete all the sessions, all the data associated with it was removed from the study. This happened for two animals that lost the headpieces before completion, resulting in a final sample size of six rats in each study. No experiments were replicated. The drug dosages were informed by previous studies.^27, 31, 32^ See Figure 1a for schematic representation of the study design.

### PPI measurement

The PPI was measured using an SR-Lab acoustic startle chamber (San Diego Instruments). The sessions consisted of pulse-alone trials (115 dB startle pulse of 40 ms duration) interspersed with prepulse+pulse trials (78 dB pulse of 20 ms preceding the startle pulse by 100 ms—background 70 dB). Within a given session, 50% of trials were pulse-alone trials and the other 50% were prepulse+pulse trials, presented in a pseudorandom order with an average intertrial interval of 15 s. At the beginning of each session, the animals were connected to the EEG equipment and placed in a clear plexiglass cylinder (internal diameter: 9 cm) with an accelerometer attached to its base. The startle responses for each trial were recorded and percentage PPI (%PPI) calculated.

### EEG acquisition

EEG was acquired using a Powerlab A-D converter and bioamplifiers (ADInstruments, Bella Vista, NSW, Australia) using Chart V 4.5 Software (ADInstruments). The sampling frequency was set at 2000 Hz. Electrical noise (50 Hz) emanating from the power mains was controlled using selective eliminators (Humbugs; Digitimer, Letchworth Garden City, UK). Throughout the session, raw electrocorticography (ECoG) was continuously recorded from both the hemispheres. Although electrophysiological analysis was performed on each channel, for each session, the data from both the channels were averaged and used for all the subsequent analyses. A third channel was connected to the PPI amplifier to precisely record the pulse and prepulse onset.

### Analysing oscillations

To assess oscillatory activity, the raw ECoG signal was processed using MATLAB scripts (v7.10.0, Natick, MA, USA; The MathWorks, 2010). We extracted two primary outcomes from the data: ongoing oscillatory activity and evoked oscillatory activity elicited by the prepulse stimuli. For the measurement of ongoing oscillations, the continuous ECoG was sectioned into 2-s epochs and each epoch was subjected to Fast Fourier Transforms using the MATLAB pwelch function, in order to calculate average power over the frequency range 1–200 Hz. For each 1-min block, average power values in the gamma (30–80 Hz) frequency band were calculated. The values obtained during the 15 min baseline period (pre-injection) were averaged for each recording and all the subsequent values for a given trial were expressed as a percentage of this baseline value (see Figure 1b for schematic illustration).

For the measurement of evoked oscillatory responses, the ECoG was subjected to time–frequency analysis to assess evoked oscillations specifically triggered by the auditory prepulse. We specifically focussed on the prepulse as we have previously shown this correlates with behavioural performance.^23^ We set the onset of the prepulse at *t*=0. Single trial epochs (−400 to +600 ms) for every prepulse+pulse trial were extracted from the continuous ECoG data and subjected to morlet wavelet decomposition using EEGlab to calculate event-related spectral perturbation at 180 linearly spaced frequencies from 20 to 200 Hz with wavelet cycles increasing from 3 to 10. Prepulse-evoked gamma activity was calculated by averaging all the values between 30 and 80 Hz occurring 0–100 ms relative to the onset of the prepulse (that is, immediately before the startling pulse) following the administration of NMDAr antagonists and expressed in dB (10log_10_ (μV^2^)). To isolate the true evoked power, this value was then ‘baseline-corrected' by subtracting the average gamma power generated from the period 300–0 ms relative to the prepulse (see Figure 1c for schematic illustration).

### Code availability

The Matlab codes used in these analyses are available—contact Associate Professor Jones.

### Drugs

Clozapine (Sigma, St. Louis, MO, USA) and olanzapine (AbcamBiochemicals, Sapphire Biosciences, Redfern, NSW, Australia) were dissolved using pure acetic acid, diluted to make a 10% acetic acid solution adjusted to pH 6.0. NFPS (AbcamBiochemicals) was dissolved in a 10% solution of 2-hydroxypropyl-β-cyclodextrin. d-serine (Sigma), haloperidol (Sigma), LY379268 (AbcamBiochemicals), ketamine hydrochloride (racemic mixture, Troy Laboratories, Glendenning, NSW, Australia) and MK-801 (Sigma) were all dissolved in 0.9% saline.

### Statistical analyses

Our primary analysis was designed to assess whether behavioural or electrophysiological deficits induced by NMDAr antagonists were reversed by the antipsychotic drugs. As such, analysis of variance with repeated measures were used for all outcome measures, and bonferroni *post hoc* planned comparisons used where appropriate. The sample sizes were chosen based on previous studies from our lab looking at identical end points. All outcome measures were objectively generated by PPI software or MATLAB scripts, and as such, scientists were not blinded to the treatment groups. All the statistical analyses were performed using GraphPad Prism. The data represent mean±s.e.m. of *n*=6 animals per group.

## Results

### Only clozapine attenuates NMDAr antagonist-induced disruptions to sensorimotor gating

**Figure 2:**
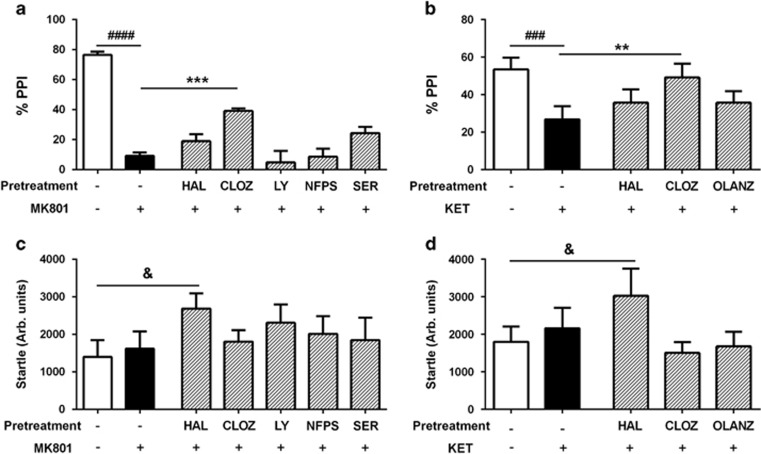
Effects of drugs on PPI and startle. MK801 (**a**) and ketamine (KET, **b**) both impaired PPI of the acoustic startle response, and this effect was significantly reduced by pretreatment with clozapine (CLOZ), but not by the other test drugs including haloperidol (HAL), LY279368 (LY), NFPS or d-serine (SER). Compared with vehicle+vehicle, the magnitude of the acoustic startle was significantly enhanced by the HAL+MK801 (**c**) and HAL+KET (**d**) conditions, but not other drug combinations. ^**###**^*P*<0.001, ^**####**^*P*<0.0001 represent the significant difference between vehicle and NMDAr antagonist conditions; **P<0.01, ***P<0.001 represent the significant difference between test drug+NMDAr antagonist and vehicle+NMDAr antagonist. ^&^*P*<0.05 represents the significant difference between vehicle+vehicle and HAL+NMDAr antagonist conditions. NMDAr, *N*-methyl-d-aspartate receptor; PPI, prepulse inhibition.

In the first study using MK801, one-way analysis of variance revealed significant differences in PPI between the treatment groups (F_(6,30)_=35.98, *P*<0.0001; Figure 2a). *Post hoc* comparisons identified a robust difference between control vs MK-801+vehicle pretreatment (*P*<0.0001). The primary objective of this study was to determine whether the antipsychotic drugs were able to impact this disruption caused by MK801. Clozapine pretreatment significantly enhanced the PPI levels, partially preventing the deficit induced by MK801 (*P*<0.001). The pretreatment with either haloperidol, LY379268, NFPS or d-serine did not impact the MK801-induced PPI deficit (*P*>0.05), identifying a unique effect of clozapine.

The second study utilized another NMDAr antagonist, ketamine, to disrupt PPI. Again we identified significant effects of treatment on PPI (F_(4,20)_=8.183, *P*=0.0004; Figure 2b), and *post hoc* analysis identified a robust effect of ketamine to disrupt this measure (*P*<0.001). When assessing the effects of pretreatment, clozapine significantly attenuated the PPI deficit induced by ketamine (*P*<0.01), an effect that was not seen with either haloperidol or olanzapine (*P*>0.05).

We also found significant differences in the startle response between the groups in both studies (MK801 study: F_(6,30)_=2.67, *P*=0.034; ketamine study: F_(4,20)_=4.74, *P*=0.008, Figures 2c and d). *Post hoc* analyses revealed that the startle response was significantly greater (*P*<0.05) following the treatment with haloperidol+NMDAr antagonist compared with vehicle treatment in both the studies.

### Only clozapine reverses NMDAr antagonist-induced evoked gamma power deficits

**Figure 3:**
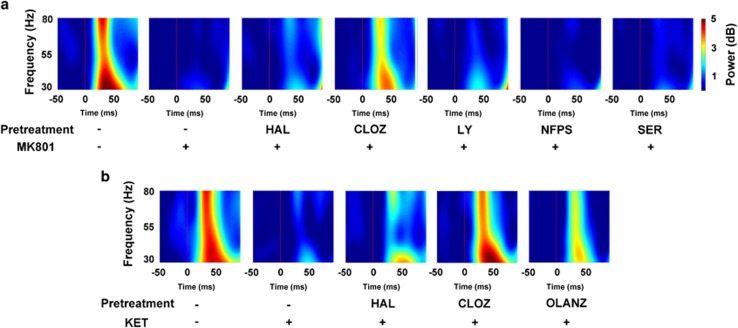
Effects of drugs on evoked electrophysiological responses measured during the prepulse inhibition session. Images represent time–frequency heat maps showing mean spectral power generated in all prepulse trials post NMDAr antagonist injection from the different conditions in the MK801 experiment (**a**) and in the ketamine experiment (**b**). The thin red vertical line on each plot at 0 ms represents the initiation of the prepulse. CLOZ, clozapine; HAL, haloperidol; LY, LY279368; NFPS, sarcosine; NMDAr, *N*-methyl-d-aspartate receptor; OLANZ, olanzapine; SER, d-serine.

**Figure 4:**
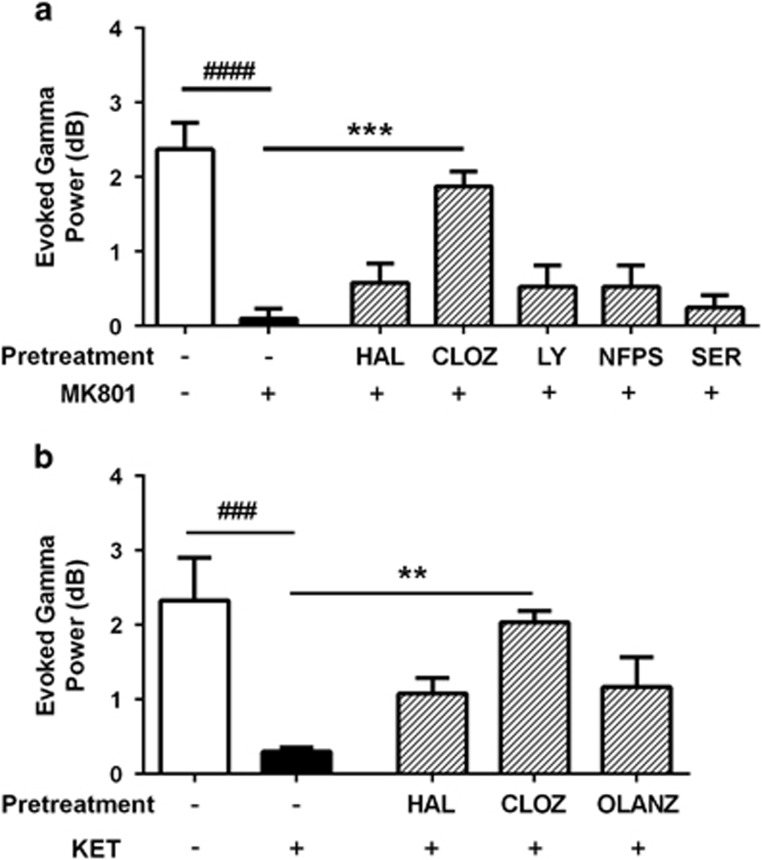
Effects of drugs on evoked gamma power measured during the behavioural task. MK801 (**a**) and KET (**b**), both markedly reduced evoked gamma power (30-80Hz), compared with vehicle treatment, and this effect was significantly blocked by pretreatment with CLOZ, but not any of the other test drugs. ^**###**^*P*<0.001, ^**####**^*P*<0.0001 represent the significant difference between vehicle and NMDAr antagonist; **P<0.01, ***P<0.001 represent the significant difference between test drug+NMDAr antagonist and vehicle+NMDAr antagonist. CLOZ, clozapine; HAL, haloperidol; KET, ketamine; LY, LY279368; NFPS, sarcosine; NMDAr, *N*-methyl-d-aspartate receptor; OLANZ, olanzapine; SER, d-serine.

Next we studied electrophysiological outcomes occurring during the PPI session. First we mapped the spectral power occurring during the prepulse trials. The spectrograms illustrating the consequence of the auditory pulses on electrophysiological activity, and the influence of treatment, are depicted in Figure 3. We quantified the oscillatory power evoked by the prepulse occurring in the gamma frequency range (30–80 Hz) and found that, in the MK801 experiment, there were significant differences between the groups when comparing evoked gamma power (F_(6,30)_=14.13, *P*<0.0001; Figure 4a). *Post hoc* analyses revealed that MK801 induced a significant reduction in power, compared with the vehicle condition (*P*<0.0001), and that clozapine pretreatment prevented this electrophysiological deficit (*P*<0.001 compared with MK801). Neither haloperidol, LY379268, NFPS or d-serine impacted the effect of MK801 on evoked power (*P*>0.05).

When assessing the ketamine experiment, we also identified a significant effect of treatment on evoked gamma power (F_(4,20)_=7.16, *P*=0.001; Figure 4b). Ketamine significantly reduced evoked power, compared with vehicle (*P*<0.001), and this was prevented by pretreatment with clozapine (*P*<0.01 compared with ketamine), but not haloperidol or olanzapine (*P*>0.05). These data bear a striking resemblance to the unique inhibitory effects of clozapine on NMDAr antagonist-induced deficits in PPI.

### Variable effects of antipsychotic drugs on NMDAr antagonist-induced elevation in ongoing gamma oscillations

**Figure 5:**
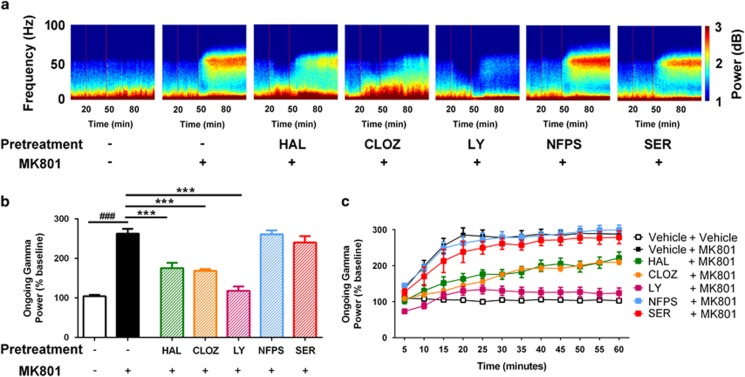
Effects of drugs on ongoing electrophysiological activity in the MK801 experiment. (**a**) Heat maps of drug effects on spectral power over time. The two red vertical lines represent the injection of the test drug at *t*=20, and of MK801 at *t*=45 min. (**b**) Quantification of the effects of drugs on gamma power following the injection of MK801, relative to the pre-injection period. MK801 induced a marked increase in ongoing gamma power, and this was significantly blunted by pretreatment with HAL, CLOZ and LY, but not affected by NFPS or SER. (**c**) These effects are also evident when visualizing the effect of pretreatments over the time course of the experiment. ^###^*P*<0.001 represents the significant difference between vehicle and MK801; ***P<0.001 represents the significant difference between test drug+MK801 and vehicle+MK801 conditions. CLOZ, clozapine; HAL, haloperidol; LY, LY279368; NFPS, sarcosine; SER, d-serine.

We then assessed the effects of the drugs on spontaneous (ongoing) gamma oscillations following injection of NMDAr antagonists. The ongoing spectrograms obtained from the MK801 study are depicted in Figure 5a. When comparing the mean gamma power over the post-injection period, we identified significant differences between the groups (F_(6,30)_=36.41, *P*<0.0001; Figure 5b). As expected, MK801 significantly elevated the power of ongoing oscillations, compared with vehicle control (*P*<0.0001). When assessing the effects of the pretreatments, we found that haloperidol (*P*<0.0001), clozapine (*P*<0.0001) and LY379268 (*P*<0.0001), all significantly reduced the effect of MK801 on ongoing power. However, neither NFPS nor d-serine had any effect (*P*>0.05). This was also evident when we assessed this measure over time (Figure 5c).

**Figure 6:**
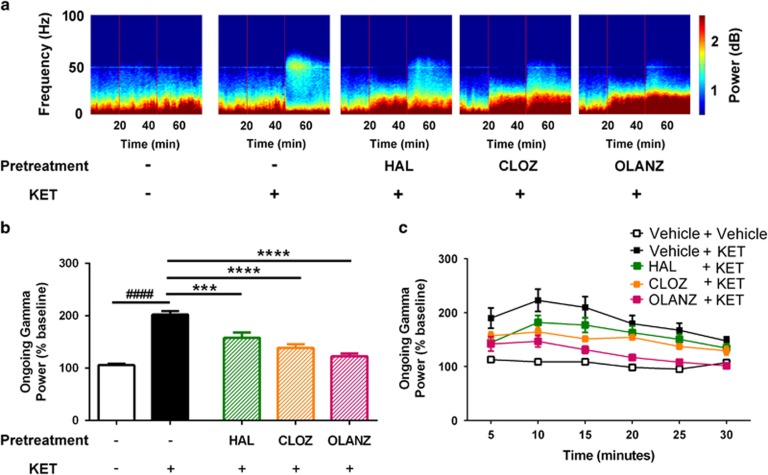
Effects of drugs on ongoing electrophysiological activity in the ketamine experiment. (**a**) Heat maps of drug effects on spectral power over time. The two red vertical lines represent the injection of the test drug at *t*=20, and of ketamine at *t*=45 min. Quantification of the effects of drugs on average gamma power following the injection of ketamine, relative to the pre-injection period (**b**). Similar to the MK801 study, ketamine elevates ongoing gamma power, but this was significantly reduced by pretreatment with HAL, CLOZ and OLANZ. (**c**) These effects are also evident when visualizing the effect of pretreatments over the time course of the experiment. ^####^*P*<0.0001 represents the significant difference between vehicle and ketamine; ***P<0.001, ****P<0.0001 represent the significant difference between test drug+ketamine and vehicle+ketamine groups. CLOZ, clozapine; HAL, haloperidol; KET, ketamine; OLANZ, olanzapine.

Comparison of the mean gamma power in the ketamine study also identified significant differences between the groups (F_(4,20)_=42.10, *P*<0.0001; Figure 6b). Ketamine enhanced gamma power compared with vehicle (*P*<0.0001), and this effect was significantly abrogated by haloperidol, clozapine and olanzapine (all *P*<0.0001 compared with ketamine). Figure 6c shows this effect over the time course of the study, further illustrating the common effects of the antipsychotics on ketamine-induced ongoing gamma power. In contrast to the evoked responses, these data bear little consistency with the effects of the pretreatments on NMDAr antagonist-induced deficits in PPI.

### Effects of repeated NMDAr antagonist exposure

Our repeated-measures design required the animals to undergo several sessions incorporating repeated exposure to NMDAr antagonists. This paradigm itself has been reported to influence many aspects of behaviour. To assess whether repeated injections systematically altered behaviour or electrophysiology over time, we compared the data generated from the pre-injection periods. We found no differences across time in PPI (F_11,55_=1.19, *P*=0.31), evoked gamma power (F_11,55_=1.12, *P*=0.36) or ongoing gamma activity (F_11,55_=0.49, *P*=0.90; Supplementary Figure 1). When analysing the data from the ketamine study, we found a similar lack of change over time in PPI (F_5,20_=1.74, *P*=0.21), evoked gamma power (F_5,20_=0.55, *P*=0.62) or ongoing gamma activity (F_5,20_=1.1, *P*=0.37). This suggests that these measures are not affected by repeated exposure to the apparatus, or to repeated injection of NMDAr antagonists.

## Discussion

In addition to producing behavioural disturbances with relevance to schizophrenia, NMDAr antagonists produce gamma oscillatory perturbations in rodents, which are reminiscent of those reported in the disorder. In the current study, we assessed the ability of antipsychotic drugs to combat deficits in gamma oscillatory activity and sensorimotor gating ability induced by NMDAr antagonists, to explore the relationship between these electrophysiological and behavioural measures. Specifically, considering PPI is sensitive to attenuation with antipsychotic drugs,^33^ we sought to assess whether antipsychotics could also combat ongoing and sensory-evoked gamma oscillatory abnormalities induced by NMDAr antagonists, and whether this was associated with their ability to normalize PPI behaviour. If disturbances of gamma oscillatory activity mediate NMDAr antagonist-induced reductions to PPI, then pharmacological reversal of gamma abnormalities should be accompanied by an improvement in behaviour. The results of this study supported this notion, with the atypical antipsychotic clozapine preventing evoked gamma activity and PPI (at least partially) following the treatment with both ketamine and MK-801. Other tested compounds were not able to influence evoked gamma abnormalities, and, perhaps consequently, did not impact the PPI deficits. Our data suggest a close relationship between evoked gamma deficits and sensorimotor gating impairment produced by NMDAr antagonists, and supports the notion that targeting-evoked gamma deficits in schizophrenia may improve cognitive symptoms.

Electrophysiological biomarkers of brain function hold great promise for schizophrenia and other brain diseases as tools to aid the diagnosis, prognosis and translation from animal models to patients. The gamma oscillations are readily measurable across species, and deficits in these oscillations in patients with schizophrenia and animal models provides a potential tool to understand how these relate to behaviour in both health and disease. Evoked gamma responses that are phase-locked to sensory stimuli are thought to be important for the early stages of sensory processing.^34^ In healthy subjects, transient elevations in gamma activity are associated with a range of basic sensory processes, including sensory gating,^35^ as well as higher order cognitive processes such as attention and working memory.^10, 11^ The reductions in auditory-evoked gamma oscillations in schizophrenia patients is one of the most consistent gamma oscillatory abnormalities reported in the disorder,^17, 36, 37, 38, 39^ and some have identified associations between auditory-evoked gamma deficits and various aspects of symptomology, particularly sensory and cognitive deficits.^40, 41^ We previously used a pharmacological model to correlate changes in evoked gamma oscillations occurring during a sensorimotor gating task with subsequent behavioural performance.^23^ The current study extends this and demonstrates that gamma oscillations that are stimulated by a behavioural task can be used as biomarkers predictive of antipsychotic-like activity, at least for sensorimotor gating behaviour. Clozapine, the atypical antipsychotic, was the only drug tested that was found to prevent disruptions to behaviour and evoked oscillations, effects which may be relevant to its unique spectrum of clinical efficacy. The fact that the other compounds tested were not able to affect NMDAr antagonist-induced disruptions to PPI or NMDAr antagonist-induced disruptions to evoked gamma oscillations, strengthens this proposition. It is curious why olanzapine, also an atypical antipsychotic, had no beneficial effect on PPI, as other studies have demonstrated restriction of NMDAr antagonist-induced PPI deficits with this compound.^42^ Olanzapine possesses a pharmacological profile similar to that of clozapine, and has been demonstrated to be equally effective in treatment-resistant schizophrenia patients,^43^ including improving cognitive symptoms.^44^ We used only one dose of olanzapine (5 mg kg^−1^, intraperitoneally), a dose which has been shown to be effective against PPI disruptions in several other reports,^45^ but perhaps this was insufficient, as others demonstrate effective reversal of PPI deficits only at higher doses.^42^ We did, however, observe effective suppression of ongoing gamma activities at this dose, which we have previously associated with PPI deficits.^23^ Or there may be biological explanations for the lack of effect of olanzapine: reports suggest clozapine may have improved efficacy compared with olanzapine specifically against attentional deficits in patient studies (for review, see refs 46, 47), and although similar, it does not have quite the extensively broad pharmacological spectrum as clozapine, possessing lower affinity at D_4_, but higher at D_2_ and α_2_ receptors.^48^ Precisely how this subtly different pharmacology may result in contrasting effects of olanzapine and clozapine here is unclear, but importantly, olanzapine did not impact either PPI or evoked gamma oscillation deficits induced by ketamine, reinforcing that behavioural improvement is closely linked to the electrophysiological normalization.

Although pharmacological attenuation of evoked gamma activity predicted improvements in PPI, this was not the case for measures of ongoing gamma power. The clinical antipsychotics haloperidol, clozapine and olanzapine, as well as the mGluR_2/3_ agonist LY379268, all significantly reduced the elevation in ongoing gamma power produced by NMDAr antagonism, which agrees with previous literature.^49, 50^ Yet, with the exception of clozapine, none of these compounds affected PPI deficits, which dissociates ongoing oscillations from having an active role in sensorimotor gating behaviour, although other behaviours, such as locomotor activity,^50^ may still be mechanistically related to ongoing oscillations. Interestingly, it has been previously suggested that the reductions in sensory-evoked gamma activity produced by NMDAr antagonists are directly related to the excessive level of ongoing gamma activity.^21^ The idea behind this concept is that the increase in ongoing gamma activity represents electrophysiological ‘noise', which impairs the ability to discriminate any transient stimulus or task-associated elevations in gamma oscillatory activity. Our data argue against this, as antipsychotic-induced reductions in ongoing gamma power were not necessarily accompanied by an increase in evoked gamma power. LY379268, for example, produced a robust reduction in the MK-801-induced rise in ongoing gamma power, such that levels of ongoing gamma activity were comparable to baseline levels, yet this compound did not increase evoked gamma power.

When considering translation of our findings, one should consider our study design. As with most acute psychopharmacology studies, we administered the antipsychotics before the disrupting NMDAr antagonists, and demonstrated that, for some outcomes, the disturbances produced by these drugs were prevented. Such drug effects may not produce the same phenotype when used against a chronically diseased brain, such as in schizophrenia. However, there is evidence to support the idea that clozapine may indeed be able to improve PPI and electrophysiological deficits in such a scenario. For example, in neurodevelopmental models of schizophrenia, such as the neonatal ventral hippocampal lesion model that exhibits PPI deficits^51^ and also electrophysiological abnormalities,^52^ clozapine has been shown to reverse PPI deficits.^53^ Although to our knowledge, auditory-evoked gamma oscillations have not been examined in this model, one might predict, based on our data, that evoked oscillations are also impaired in the model and reversed by clozapine.

An unexpected observation from our study was the ineffectiveness of the preclinical compounds, LY379268, NFPS and d-serine, which were developed to directly overcome NMDAr hypofunction, on gamma oscillations and sensorimotor gating deficits. The doses, and drugs, were chosen based on the previous reports of positive effects using models of schizophrenia. NFPS, which inhibits the synaptic uptake of the NMDAr co-agonist glycine, and d-serine, a compound which can stimulate the glycine site, both improve MK801-induced deficits in the novel object recognition task^54^ but had no effects on any outcomes here. The mGluR_2/3_ receptor agonist LY379268 reduces the effects of NMDAr antagonism on ongoing gamma oscillations and locomotor activity,^27, 55^ and reduces excessive glutamate release caused by ketamine,^28^ but was without effect on evoked oscillations or PPI. Although it should be considered that the doses of these compounds were not optimal, this suggests that different circuit mechanisms are driving the NMDAr antagonist effects on evoked gamma oscillations/PPI compared with ongoing gamma oscillations, glutamate release and locomotor activity.

One element which needs to be considered with repeated behavioural measurement is habituation to the procedure, or to sensitization of drug effects. We were able to demonstrate that there was no consequence of repeated testing, or repeated treatment with NMDAr antagonists, on any of the outcome measures, as the baseline values did not change over time. However, we cannot make any conclusions about whether sensitization to NMDAr antagonists occurred. This may be important, as others have demonstrated that subchronic exposure to ketamine can impact baseline PPI over time. It is unlikely that this influences our conclusions because as the antipsychotics were given in a random order, any sensitization of drug effect would result in increased variability of our outcome measures rather than a systematic bias.

With respect to the amplitude of the startle response itself, we found that the combination of haloperidol+NMDAr antagonist slightly, but significantly, elevated startle. This was unexpected and does not agree with the majority of literature, which show no effects or a suppression of startle with haloperidol treatment.^53^ However, one study showed that different strains of mice respond differently to haloperidol, with c57Bl6 mice showing enhanced startle, but no effect in DBA mice.^56^ It may be an unusual combination of genes in our Wistar rats, which resulted in our findings, but it is comforting that this effect was observed in both cohorts of rats.

In summary, our results suggest a close relationship between evoked gamma oscillations and sensorimotor gating ability in the NMDAr antagonist animal model. It is of interest that clozapine, the only compound capable of limiting the disruptions to evoked gamma activity and PPI in this model, also displays a unique clinical profile: clozapine is considered to be the most effective antipsychotic in cases of treatment-resistant schizophrenia,^57^ and numerous studies demonstrate the ability for clozapine to enhance cognition to some extent in patients with schizophrenia,^58, 59, 60^ although this is not a consistent observation across the literature. Considering that gamma oscillatory abnormalities have been suggested to be particularly relevant to the treatment-resistant symptoms of schizophrenia,^41^ the ability for clozapine, but not other antipsychotic agents, to normalize evoked gamma activity in the NMDAr antagonist model may have some relevance to the unique clinical effects of this drug. This study highlights the utility of evoked high frequency oscillations as predictive biomarkers of antipsychotic activity which are readily translatable across species. These oscillations deserve further characterization in patients with schizophrenia, and developed as clinical outcomes for testing antipsychotic efficacy.
