# Supplementary figures and images for: Reversal of evoked gamma oscillation deficits is predictive of antipsychotic activity with a unique profile for clozapine
Source: Transl Psychiatry. 2016 Apr 19;6(4):e784–. doi: 10.1038/tp.2016.51 (PMC4872409; doi:10.1038/tp.2016.51)

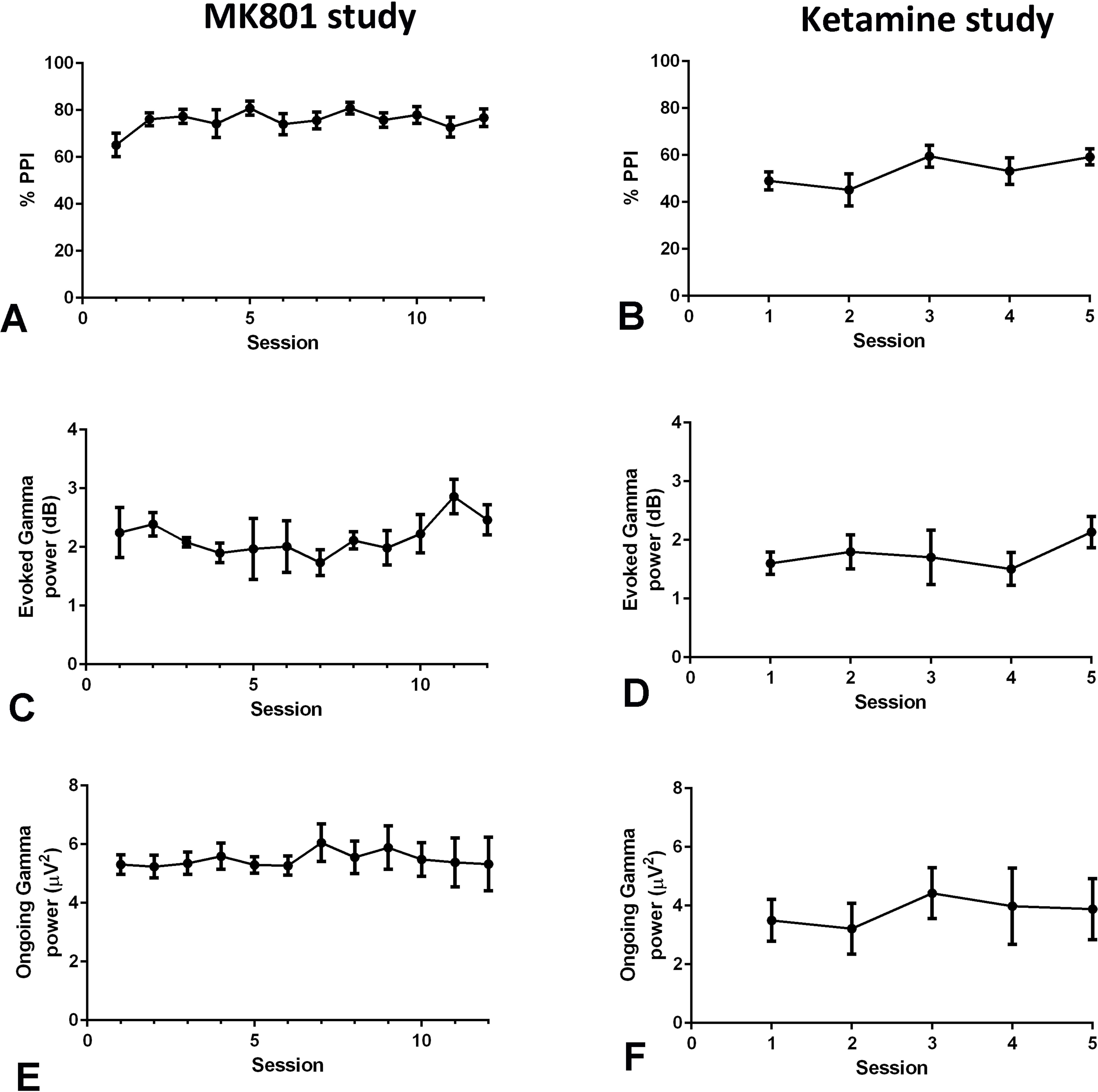

Supplement: Supplementary Figure 1 [file tp201651x1.tif]
