# Supplementary material for: Reversal of evoked gamma oscillation deficits is predictive of antipsychotic activity with a unique profile for clozapine
Source: Transl Psychiatry. 2016 Apr 19;6(4):e784–. doi: 10.1038/tp.2016.51 (PMC4872409; doi:10.1038/tp.2016.51)
Supplement: Supplementary Figure 1 Legend [file tp201651x2.doc]

**Supplementary Figure 1:** Sensorimotor behaviour (A, B), and evoked (C, D) and ongoing gamma oscillations (E, F) remain stable over consecutive sessions in both the MK801 and ketamine studies. No significant effect of time is noted with these measures.
